# Salvage Endoscopic Skull Base Surgery: Another Treatment Option After Immunotherapy for Recurrent Nasopharyngeal Carcinoma

**DOI:** 10.3389/fimmu.2022.899932

**Published:** 2022-05-24

**Authors:** Zhouying Peng, Yumin Wang, Yan Fang, Yaxuan Wang, Xiaotian Yuan, Mingxia Shuai, Shumin Xie, Ruohao Fan, Hua Zhang, Zhihai Xie, Weihong Jiang

**Affiliations:** ^1^ Department of Otolaryngology Head and Neck Surgery, Xiangya Hospital, Central South University, Changsha, China; ^2^ Otolaryngology Major Disease Research Key Laboratory of Hunan Province, Changsha, China; ^3^ National Clinical Research Center for Geriatric Disorders, Xiangya Hospital, Central South University, Changsha, China; ^4^ Anatomy Laboratory of Division of Nose and Cranial Base, Clinical Anatomy Center of Xiangya Hospital, Central South University, Changsha, China

**Keywords:** advanced recurrent nasopharyngeal carcinoma, immunotherapy, PD-1, salvage endoscopic skull base nasopharyngectomy, combination

## Abstract

**Background:**

Advanced recurrent nasopharyngeal carcinoma (NPC) is a relatively common nasopharyngeal skull base disease for which there is no uniform treatment modality. Not all patients are satisfied with the efficacy of immunotherapy with or without chemotherapy.

**Methods:**

This study included patients who underwent salvage endoscopic skull base nasopharyngectomy after immunotherapy between February 2017 and June 2021. Patient survival information was analyzed. Relevant publications were retrieved from five databases from December 1, 2011 to December 1, 2021. The outcomes of patients with advanced recurrent NPC who received programmed death 1 (PD-1) immunotherapy were collected and analyzed.

**Results:**

Nine patients who underwent skull base surgery, all of whom had previously undergone PD-1 immunotherapy, were included in this study. The 2-year overall survival (OS) and progression-free survival (PFS) rates of these patients were 25% and 29.2%, respectively. Eight publications involving 688 patients with advanced recurrent NPC were also included in this study. The combined complete response (CR), partial response (PR), and stable disease (SD) values were 2%, 23%, and 29%, respectively. The combined DCR included the three disease conditions, CR, PR, and SD, with a value of 53%. PD-1 monotherapy was more effective than PD-1 combination chemotherapy.

**Conclusions:**

PD-1 immunotherapy may improve the remission rate in patients with recurrent NPC. Salvage endoscopic skull base nasopharyngectomy may be another option for patients with poor immunotherapeutic outcomes. For patients with advanced recurrent NPC, better evidence-based medical data are needed to determine whether they should receive immunotherapy before or after surgery.

## Introduction

Advanced recurrent nasopharyngeal carcinoma (NPC) is a frequently reported skull base tumor in southern China, southeast Asia, and north Africa (1–4). NPC originates in the epithelial lining of the nasopharynx and is associated with EBV infection. Primary NPC is sensitive to radiotherapy, with intensity-modulated radiotherapy (IMRT) being the most widely used technique (5, 6). Despite recent improvements in treatment techniques, approximately 10%–20% of patients with 70 Gy IMRT still experience local recurrence, which is a substantial challenge for clinicians (1, 7, 8). Immunotherapy has a role as a novel cancer treatment modality, especially immune checkpoint inhibitor (ICI) therapy. The clinical study of programmed death 1 (PD-1) has made a breakthrough in the treatment of advance recurrent NPC (9–12). However, a review of these studies revealed that the control rate of immunotherapy for advanced recurrent NPC is limited. The general complete response (CR) rate of ICI therapy was 1.3%–16.7%, the partial response (PR) rate was only 12.2%–32%, the stable disease (SD) rate was 19.5%–51.9%, and the 1-year progression-free survival (PFS) rate was 19.3%–33.4% (13, 14). In addition, systemic adverse events associated with immunotherapy are intolerable in many patients with advanced disease.

Skull base surgery for recurrent NPC, also called salvage endoscopic skull base nasopharyngectomy, has played an important role in the treatment of advanced recurrent NPC (1–3, 8). For patients with advanced recurrent NPC whose whole-body health status can tolerate surgery, the rT1–2 and rT3–4 2-year overall survival (OS) rates are 83.9% and 35.9%, respectively. The 2-year disease-free survival (DFS) rate is 42.6% (3). With the development of endoscopic techniques and increased familiarity of skull base surgeons with nasopharyngeal anatomy, surgery for patients with local recurrence with or without local metastasis may be another treatment option for patients with advanced recurrent NPC.

This article reviews patients with advanced recurrent NPC who underwent salvage endoscopic nasopharyngectomy at our institution. These patients received several cycles of immunotherapy after recurrence, and then underwent surgical treatment after unsatisfactory immunotherapy results. This article also summarizes the available research findings to better understand the efficacy and safety of ICI with or without chemotherapy for advanced recurrent NPC.

## Materials and Methods

### Clinical Data

#### General Information

We reviewed data from February 2017 to June 2021 regarding patients with recurrent NPC who underwent salvage endoscopic nasopharyngectomy at our institution. Nine of these patients with recurrent NPC with or without cervical lymph node metastasis had received 1–4 treatment cycles of ICI immunotherapy before surgery. All patients underwent nasal endoscopy, nasopharyngeal computed tomography (CT), skull base-enhanced magnetic resonance imaging (MRI), and whole-body positron emission tomography (PET) to show the presence of recurrent NPC, and the diagnosis was confirmed by clinical endoscopic biopsy.

The inclusion criteria for this group of patients were the presence of recurrent tumors and no significant contraindications to surgery. All surgeries were performed by Dr. Weihong Jiang at Xiangya Hospital of Central South University. Additional clinical information was collected through medical case records and follow-up.

#### Observation Indexes and Statistical Analysis

The following data were collected: age, sex, treatment at initial presentation, time to recurrence, treatment after recurrence, lymph node metastasis at recurrence, concomitant symptoms or disease at recurrence, postoperative treatment, and survival status. The follow-up period was defined as the period from the time of the first surgery at our institution to the date of death or last contact. The Statistical Package for the Social Sciences version 25.0 (IBM SPSS Statistics 25.0) was used for statistical analysis. Kaplan–Meier product-limit analysis was used to calculate the OS and PFS rates. The OS rate was calculated from the date of surgery to the date of death from any cause. PFS rate was calculated from the date of surgery to the date of disease progression or death from any cause.

### Meta-Analysis

#### Search Strategy

In this study, we conducted a literature search of several major electronic databases, including PubMed, Web of Science, Cochrane, and two Chinese databases (CNKI and Wanfang). The publications analyzed were published from December 1, 2011 to December 1, 2021. The search strategy was predefined according to the following Medical Subject Headings (MeSH) and free terms: “recurrent” or “recurrence,” “nasopharyngeal carcinoma,” “immunotherapy,” or “immune checkpoint inhibitor.” Additional relevant articles were obtained by searching the reference lists of articles included in this study. Simultaneously, the references of included papers were examined for potentially eligible studies.

#### Study Selection Criteria

Two investigators independently conducted the literature search in accordance with the following inclusion and exclusion criteria. Disagreements were resolved by discussion and consensus. Eligible studies in this meta-analysis met the following criteria: (a) study design: cohort study; (b) targeted population: advanced NPC; (c) treatment modality: ICI with or without standard chemotherapy; (d) publications included data describing treatment efficacy and description of adverse events; (e) when multiple studies reported the same sample, the most complete data from the available studies were selected. The following criteria were used to exclude studies: (a) study design: case reports, reviews, and meta-analyses, and (b) studies without complete efficacy evaluation data.

#### Data Extraction and Assessment

After retrieval, the relevant publications were screened according to their titles and abstracts, and inappropriate publications were removed. Finally, the remaining publications were thoroughly reviewed for their final inclusion in this study. Two researchers read all retrieved publications simultaneously and independently. In the case of disagreement between the two researchers, a third researcher was required to intervene and discuss to reach a unified opinion. Finally, valid data were extracted from the publications that met the inclusion criteria. The extracted information included the sample characteristics, specific information on treatment, post-treatment follow-up time, and survival rate.

#### Statistical Analysis

We analyzed the obtained data for the survival rate, PR, CR, SD, and other factors using Review Manager 5.4.1. The rate values were merged, and heterogeneity tests were performed. *I* ^2^ > 50% was defined as significant heterogeneity, and a random-effects model was used to merge the rate values with the 95% CI. **Supplementary Figure 1** for publication bias analysis.

## Results

### Clinical Cohort

Nine patients meeting the criteria for recurrent NPC were included in this cohort, all of whom underwent salvage endoscopic nasopharyngectomy after unsatisfactory immunotherapy results. The mean age of the patients was 48.2 years (range 32–57). There was a high proportion of male patients in this group (66.7%). The other clinical characteristics of the patients in this group are shown in **Table 1**.

**Table 1:** Demographics and clinical characteristics of patients with recurrent NPC in this study.

Characteristics	Total = 9	%
**Gender**		
Male	6	66.7
Female	3	33.3
**Age (years)**		
≥45	6	66.7
<45	3	33.3
**Interval to recurrence**		
≥3 years	7	77.8
<3 years	2	22.2
**Initial lymph node metastasis**		
Yes	4	44.4
No	5	55.6
**Recurrent T staging**		
rT1–2	1	11.1
rT3–4	8	88.9
**Immunotherapy cycle**		
≥3 cycles	2	22.2
<3 cycles	7	77.8
**Immunotherapy response**		
PR	3	33.3
SD	2	22.2
PD	4	44.4
**Tumor encircling ICA**		
Yes	4	44.4
No	5	55.6
**Follow-up time(months)**		
≥12	6	66.7
<12	3	33.3
**Outcome**		
Alive	5	55.6
Death	4	44.4

NPC, nasopharyngeal carcinoma; ICA, internal carotid artery.

**Figure 1:**
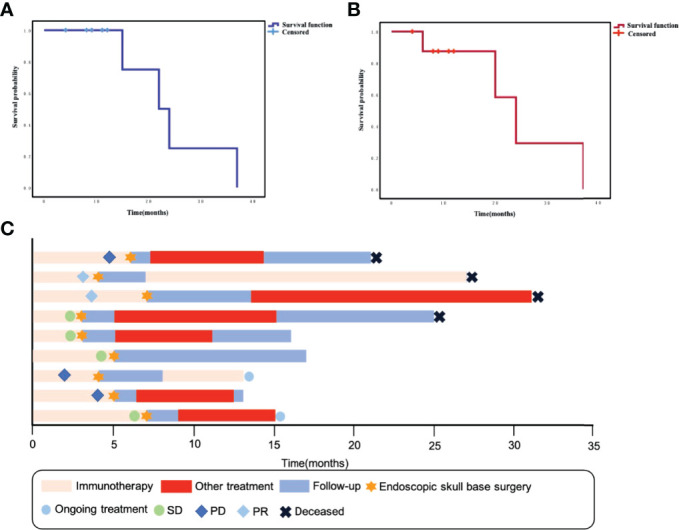
**(A)** Kaplan–Meier curve of overall survival for this group of patients. **(B)** Kaplan–Meier curve of progression-free survival for this group of patients. **(C)** Swimming plot shows the treatment process for each patient.

Eight patients in this group had rT3–4 recurrent NPC, and four had tumors that encircled the internal carotid artery (ICA). These patients underwent ICA embolization or vascular bypass surgery before salvage surgery. Only one patient with rT2 had no other postoperative treatment and was followed up periodically. The remaining patients received chemotherapy or oral chemotherapeutic agents, with or without immunotherapy after surgery. Currently, four patients have died, one due to pulmonary metastasis of the tumor and three patients due to causes without direct relation to the recurrence or metastasis of the tumor. The 2-year OS rate of these patients was 25% and the 2-year PFS rate was 29.2% (**Figures 1A, B**). Specific consultation processes and outcomes are shown in **Figure 1C**.

### Meta-Analysis

#### Search Results and Study Characteristics

**Figure 2:**
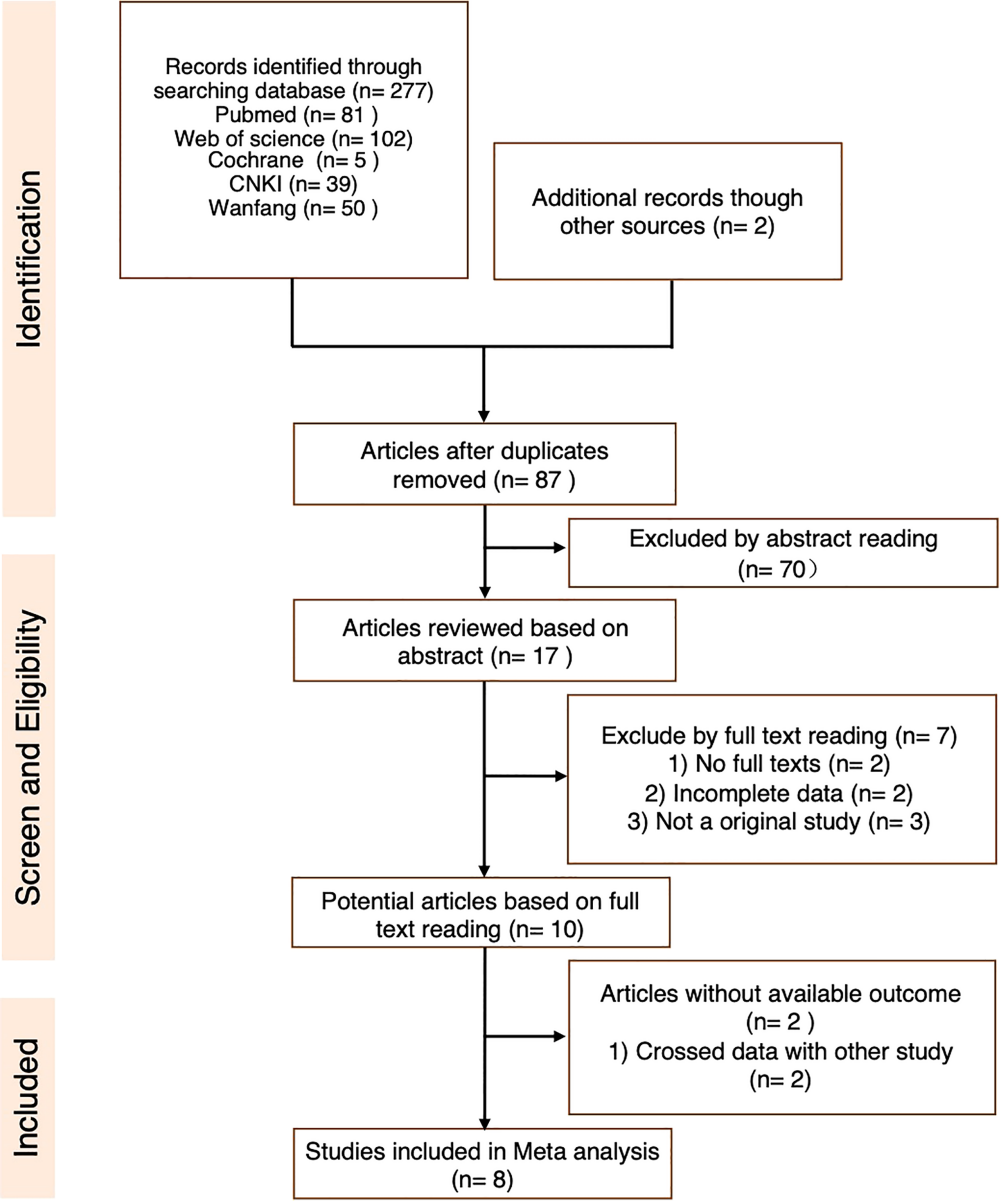
Flowchart of the process of trial selection.

An initial systematic study of the literature published between December 1, 2011 and December 1, 2021 was conducted by including eight publications. All were found in the English database. While reviewing the titles and abstracts, ten were selected for full-text reading (the specific reasons are shown in **Figure 2**). Eight studies were included in our analysis. Data from 688 patients with a follow-up duration as long as 100.2 months were pooled together. The characteristics of the included studies are shown in **Table 2**. The characteristics of the patients’ demography for this analysis are shown in **Table 3**.

**Table 2:** Characteristics of included studies.

Author	Year	Location	Inclusion period	Sample size	Anti-PD-1 drug	Design	Experimental regimen
Hsu et al. (9)	2017	Multinational	2014–2016	27	Pembrolizumab	Phase I	10 mg/kg every 2 weeks up to 2 years or until disease progression or unacceptable toxicity
Ma et al. (10)	2018	Multinational	2015–2016	45	Nivolumab	Phase I	3 mg/kg intravenously every 2 weeks on a 4-week cycle until they experienced disease progression
Fang et al. (15)	2018	Multinational	2016–2017	93	Camrelizumab	Phase I	1 mg/kg, 3 mg/kg, and 10 mg/kg, and a bridging dose of 200 mg per dose once every 2 weeks
Sato et al. (11)	2020	Japan	2017–2018	12	Nivolumab	Retrospective study	3 mg/kg or 240 mg (fixed dose)by intravenous drip infusion at 2-week intervals
Ma et al. (16)	2021	China	2016–2018	124	Camrelizumab Nivolumab	Phase I	1 mg/kg, 3 mg/kg, 200 mg or 10 mg/kg every 2 weeks; or nivolumab at dosage of 3 mg/kg, 240 mg every 2 weeks and 360 mg every 3 weeks
Jin et al. (17)	2021	China	2018–2020	41	Camrelizumab Toripalimab Penpulimab Tislelizumab	Retrospective study	200 mg on day 1 every 2 or 3 weeks, toripalimab (240 mg) on day 1 every 3 weeks
Wang et al. (12)	2021	China	2016–2019	190	Toripalimab	Phase II	3 mg/kg toripalimab once every 2 weeks *via* intravenous infusion until confirmed disease progression or unacceptable toxicity.
Yang et al. (4)	2021	China	2018–2019	156	Camrelizumab	Phase II	200 mg intravenously every 2 weeks

**Table 3:** Characteristics of patients’ demography and clinical endpoints.

Author	Age, median (range), years	Gender, male, *n* (%), female, *n* (%)	PD-L1 expression		Clinical endpoints		Clinical response			1-year PFS rate (%)	Median follow-up, months
			<1%, (%)	≥1%, (%)	Primary endpoint	Secondary endpoint	CR (%)	PR (%)	SD (%)		
Hsu et al., 2017 (9)	52 (18–68)	21 (77.8)	0	100.0	ORR, PR	PFS,OS, DOR	0	25.9	51.9	33.4	20.0 (2.2–26.8)
		6 (22.2)									
Ma et al., 2018 (10)	57 (37–76)	35 (77.8)	57.1	42.9	ORR	PFS,OS, DOR	2.2	17.8	33.3	19.3	12.5 (2.2–22.0)
		10 (22.2)									
Fang et al., 2018 (15)	45 (38–52)	75 (81.0)	/	/	Safety, tolerability	Antitumor activity	2.0	32.0	/	27.1	9.9 (8.1–11.7)
		18 (19.0)									
Sato et al., 2020 (11)	58 (30–67)	10 (83.0)	/	8.0	OS	PFS,ORR,DCR	17.0	0	25.0	33.0	11.9 (2.8–21.7)
		2 (17.0)									
Ma et al., 2021 (16)	46 (23–73)	95 (76.6)	/	/	ORR	DOR,PFS,OS	1.6	28.2	/	/	24.7 (23.3–26.6)
		29 (23.4)									
Jin et al., 2021 (17)	<60: 30 (73.2%)	28 (68.3)	/	/	ORR	DFS,OS	2.4	12.2	29.3	20.5	7.0 (2.0–19.0)
	≥60: 11 (26.8%)	13 (31.7)									
Wang et al., 2021 (12)	46.4 (22–71)	158 (83.2)	25.3	70.5	ORR	DFS,OS,DOR	2.6	17.9	19.5	/	/
		32 (16.8)									
Yang et al., 2021 (4)	48 (23–71)	124 (79.5)	23.1	73.1	ORR	DOR,DCR,PFS,OS	1.3	26.9	26.3	/	14.2 (0.7–27.6)
		32 (20.5)									

CR, complete response; PR, partial response; SD, stable disease; PFS, progression-free survival; ORR, objective response rate; DOR, duration of response; DCR, disease control rate; DFS, disease-free survival; OS, overall survival.

#### The Efficacy and Adverse Events of PD-1 Treatment

**Figure 3:**
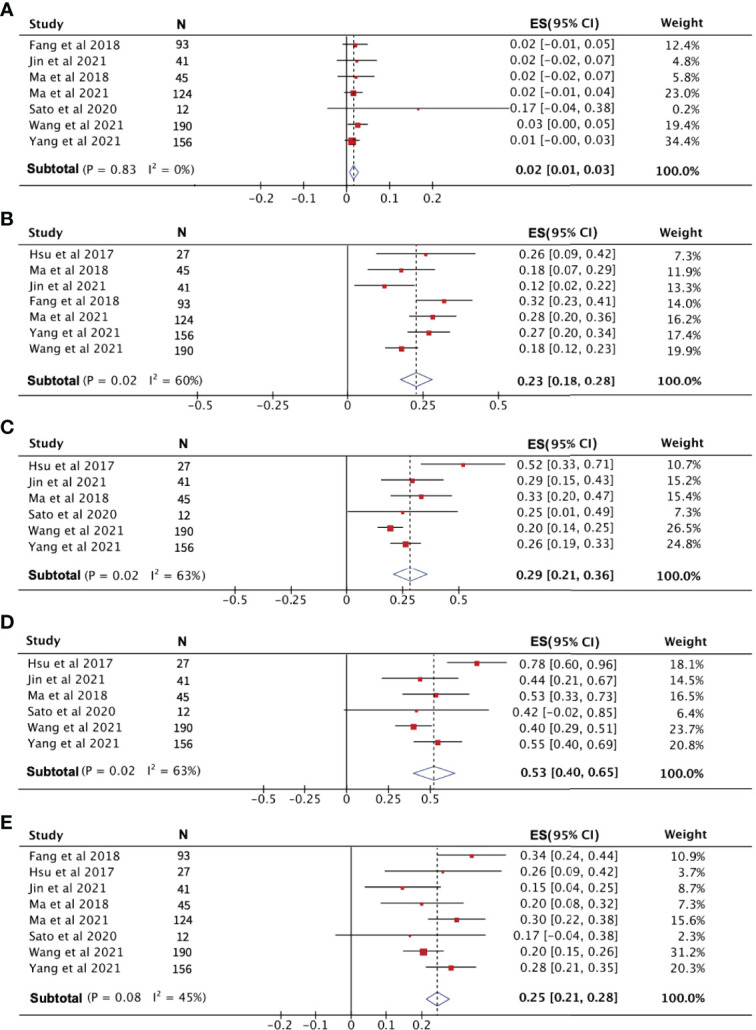
The evaluation of disease condition in patients receiving PD-1 treatment. **(A)** Complete response rate. **(B)** Partial response rate. **(C)** Stable disease rate. **(D)** Disease control rate. **(E)** Objective response rate.

**Figure 4:**
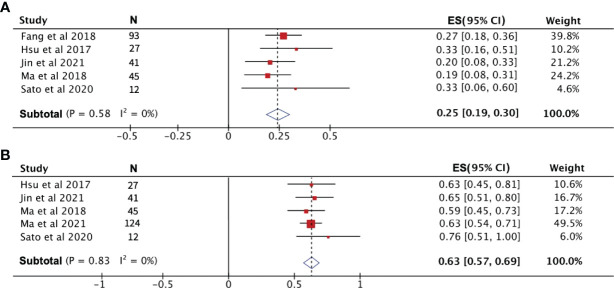
The 1-year progression-free survival rate **(A)** and overall survival rate **(B)** of patients receiving PD-1 treatment.

**Figure 5:**
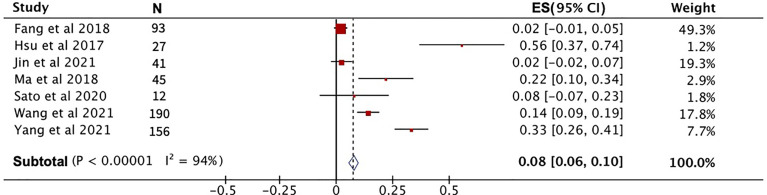
Rate of ≥ G3 adverse events in patients receiving PD-1 treatment.

After pooling the data from eight high-quality publications, the disease status of patients who received PD-1 treatment was evaluated. As shown in **Figures 3A–C**, the combined CR, PR, and SD were 2%, 23% and 29%, respectively. The combined DCR included the three disease conditions CR, PR, and SD, with a value of 53% (**Figure 3D**). The pooled ORR, indicating a combination of CR and PR rates, was 25% (**Figure 3E**). During the follow-up period, the PFS rate in patients treated with PD-1 was combined, and the 1-year PFS rate was 25% with a 95% confidence interval of 19%–30% (**Figure 4A**). The pooled 1-year OS rate was 63%, with a 95% confidence interval of 57%–69% (**Figure 4B**). The rate of adverse events occurring ≥G3 in the eight publications included in this study was combined, with an incidence of 8% (95% CI: 6%–10%), as shown in **Figure 5**.

#### Analysis of PD-1 Efficacy With or Without Chemotherapy

**Figure 6:**
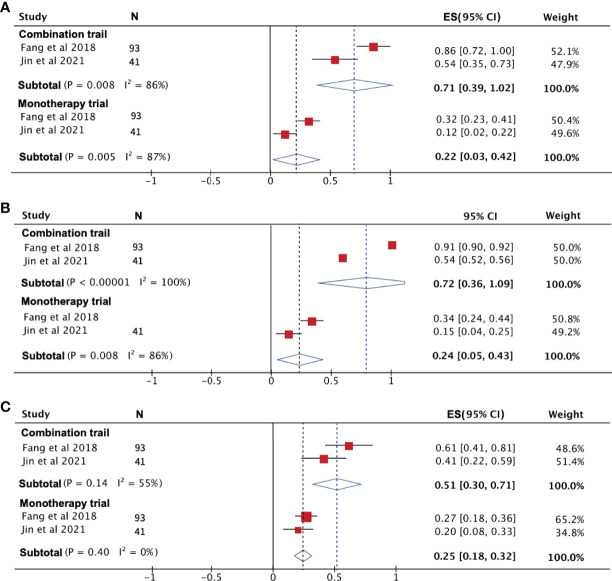
The efficacy of PD-1 treatment with or without chemotherapy. **(A)** Partial response rate. **(B)** Objective response rate. **(C)** Progression-free survival rate.

To compare the efficacy of PD-1 monotherapy versus PD-1 combination chemotherapy in recurrent NPC, we combined the PR, ORR, and 1-year PFS rates from two publications. PR and ORR were significantly higher in patients receiving combination therapy than in those receiving PD-1 monotherapy, with a PR and ORR of 71% and 72%, respectively, for PD-1 combination chemotherapy compared to only 22% and 24% for monotherapy (**Figures 6A, B**). The 1-year PFS rates for the two treatment modalities were 51% (95% CI: 30%–71%) for combination therapy and 25% (95% CI: 18%–32%) for monotherapy (**Figure 6C**).

## Discussion

Recurrence and metastasis are the main causes of treatment failure in NPC patients. With the development of radiotherapy technology, the 5-year OS rate of NPC can reach 50%–64%. However, 10%–20% of patients still develop recurrence or metastasis after the first treatment improvement (1, 18, 19). Palliative treatment is preferred for patients with recurrent NPC and distant metastases. However, there may be multiple treatment options for recurrent NPC with or without cervical lymph node metastases. Radiotherapy, as the main treatment modality for NPC, is also important for the treatment of recurrence (20). For recurrent NPC, the tumor is usually not limited to the nasopharynx. Especially in rT3–4 recurrent NPC, the bone and soft tissue at the skull base are easily invaded, and the ICA is adjacent to or even encircled by the lesion (2, 21–23). For patients whose skull base structures are affected by the tumor, the design of the target area and dose control are difficult if they receive secondary radiotherapy, and the probability of fatal complications is higher (24).

In recent years, an increasing number of publications have reported the application and efficacy of immunotherapy in advanced recurrent NPC, and clinical evidence of PD-1 inhibitors in patients with advanced recurrent NPC has been accumulated. This study summarizes the available publications on ICIs in advanced recurrent NPC, with the aim of summarizing the efficacy and safety of PD-1 inhibitors and comparing the survival of patients with and without standard chemotherapy combined with immunotherapy. It is noteworthy that patients with recurrence and distant metastases account for a relatively large proportion of published publications on the outcome of immunotherapy for advanced recurrent NPC. Although the outcomes of patients receiving immunotherapy are not generally discussed separately, empirically, the results are not better than those of patients with local recurrence. Moreover, whether there is a relationship between the T-stage of recurrent tumors and the effect of immunotherapy has not been much discussed in published papers. We also report the recent 5-year survival of patients with advanced recurrent NPC who did not achieve a satisfactory outcome after immunotherapy and ultimately opted for salvage endoscopic skull base nasopharyngectomy at our institution. This article may provide a clinical basis for choosing surgical treatment for patients with advanced recurrent NPC who have received immunotherapy but have not achieved satisfactory outcomes.

Our study covered the results from eight publications over the last 10 years, combining 688 patients followed up for 100.2 months of data. The pooled study data suggest that, for advanced recurrent NPC, PD-1 treatment reports are currently dominated by the results of phase I and II clinical trials. From the pooled results of this study, the combined CR rate was only 2%, the PR rate was 23%, and the 1-year PFS rate was 25%. This shows that the efficacy of immunotherapy remains limited in patients with advanced recurrent NPC, with or without standard chemotherapy. Salvage endoscopic skull base nasopharyngectomy as a surgical treatment has recently been increasingly used for simple recurrent NPC with or without cervical lymph node metastases. After undergoing surgical treatment, the 2-year DFS rate was 40.0%–92.8% and the 5-year OS rate was 38.3%–78.1% (1, 3, 25–27). These results indicate that surgical treatment plays an increasingly important role in the treatment of recurrent NPC. Surgical treatment has a certain probability of prolonging the survival of this group of patients without serious complications.

This study has some limitations that should be acknowledged. First, eight studies were analyzed, of which six were prospective and two were retrospective. No randomized controlled design was included in the studies. In addition, smaller studies were included in the analysis. Although the case series reported by our institution provides a small basis for future research, there are still no controlled trials confirming that surgical treatment necessarily prolongs survival in advanced recurrent NPC compared to immunotherapy. This also requires the design of larger prospective randomized controlled clinical trials in the future to provide medical evidence for the efficacy of surgical treatment. Several clinical trials are currently underway, and a single-arm phase II trial (NCT05011227) hosted by Fudan University Eye, Ear, Nose, and Throat Hospital is enrolling subjects with resectable recurrent NPC at stage rT2 or higher, attempting to compare the preoperative receiving efficacy of surgery followed by immunotherapy or chemotherapy. Whether immunotherapy or surgical treatment can prolong the survival period and improve the survival of patients has yet to be explored by designing relevant clinical trials. Our institution will also soon conduct a clinical study in patients with rT3–4 recurrent NPC treated with or without immunotherapy after salvage endoscopic skull base nasopharyngectomy in patients with tumor invasion of skull base structures and the ICA.

## Conclusion

PD-1 immunotherapy may have a remission rate in patients with recurrent NPC. However, salvage endoscopic skull base nasopharyngectomy is another option for patients with SD, PR, or progressive disease. For patients with recurrent NPC, especially those with advanced recurrence, better evidence-based medical data are still needed to determine if they should receive immunotherapy before or after surgery.

## Data Availability Statement

The raw data supporting the conclusions of this article will be made available by the authors, without undue reservation.

## Ethics Statement

The studies involving human participants were reviewed and approved by Xiangya Hospital Research Ethics Committee of the Central South University. The patients/participants provided their written informed consent to participate in this study.

## Author Contributions

WJ and ZX conceived and designed the study. HZ, RF, YuW, and ZP searched the database and calibrated the analysis results. ZP, YF, YaW, XY, and MS performed the analysis, and prepared the figures and tables. ZP wrote the main manuscript. SX corrected the manuscript. All of the authors reviewed the manuscript. All authors read and approved the final manuscript.

## Funding

This research was funded by the National Natural Science Foundation of China (82171118), the Hunan Postdoctoral Program for Innovative Talent (2021RC2017), and the Natural Science Foundation of Hunan Province (2021JJ41027). The funders had no role in study design, data collection and analysis, decision to publish, or preparation of the manuscript.

## Conflict of Interest

The authors declare that the research was conducted in the absence of any commercial or financial relationships that could be construed as a potential conflict of interest.

## Publisher’s Note

All claims expressed in this article are solely those of the authors and do not necessarily represent those of their affiliated organizations, or those of the publisher, the editors and the reviewers. Any product that may be evaluated in this article, or claim that may be made by its manufacturer, is not guaranteed or endorsed by the publisher.

## Supplementary Material

Supplementary Figure 1 Publication bias analysis. **(A)** Complete response rate. **(B)** Partial response rate. **(C)** Stable disease rate. **(D)** Disease control rate. **(E)** Objective response rate. **(F)** One-year progression-free survival rate. **(G)** Overall survival rate. **(H)** Adverse events.
