# Supplementary figures and images for: Salvage Endoscopic Skull Base Surgery: Another Treatment Option After Immunotherapy for Recurrent Nasopharyngeal Carcinoma
Source: Front Immunol. 2022 May 24;13:899932. doi: 10.3389/fimmu.2022.899932 (PMC9170997; doi:10.3389/fimmu.2022.899932)

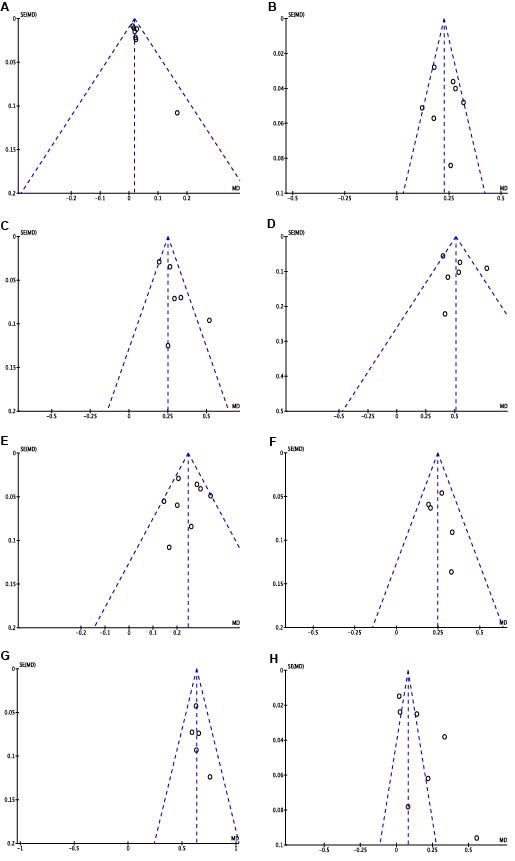

Supplement: Supplementary Figure 1 — Publication bias analysis. (A) Complete response rate. (B) Partial response rate. (C) Stable disease rate. (D) Disease control rate. (E) Objective response rate. (F) One-year progression-free survival rate. (G) Overall survival rate. (H) Adverse events. [file Image_1.jpeg]
